# Mucosal atrophy in collagenous colitis: a case report

**DOI:** 10.1186/1471-230X-11-114

**Published:** 2011-10-25

**Authors:** Michael Majores, Steffen Wunsch, Bernd Blume, Hans-Peter Fischer, Christoph Reichel

**Affiliations:** 1 Institute of Pathology, University of Bonn, Bonn Germany; 2 Rehabilitation Centre Bad Brückenau, Clinic Hartwald, German Pension Insurance Federal Office, Bad Brückenau, Germany; 3 Institute of Pathology, Schweinfurt, Germany; 4 Department of Internal Medicine I, University Clinic Bonn, Bonn Germany

## Abstract

**Background:**

Mucosal atrophy as a potential cause of impaired colonic compliance has not yet been described as a complication in Collagenous Colitis (CC).

**Case presentation:**

We present a 51-year-old female patient with a 20-year history of diarrhea and diagnosed with CC ten years prior to her presentation. We reviewed reports from three colonoscopies performed after the diagnosis. Overall 12 biopsies obtained in the last two colonoscopies were re-analyzed by two pathologists blinded to the aim of the study. Besides the typical histological findings of CC, the endoscopic appearance was normal, and no histological signs of atrophy were found during the first colonoscopy. Surprisingly, the second and third colonoscopy revealed a region of advanced segmental mucosal atrophy in the cecum with the mucosal height normalizing toward the transverse colon. This pattern of atrophy was inversely related to the pattern of sub-epithelial collagen deposition, which increased toward the rectum.

**Conclusion:**

If no chance occurrence, our observation supports the idea that additional factors, probably luminal in nature, may be co-responsible for the mucosal atrophy in this case. Thus, mucosal atrophy in the proximal colon appears to be a new candidate among the growing list of rare complications associated with long standing CC.

## Background

Collagenous colitis (CC) is a chronic inflammatory bowel disease characterized by chronic watery, non-bloody diarrhea in the context of macroscopically normal appearing colonic mucosa on endoscopic examination. CC was first described in 1976 [1]. Although it was initially underestimated, it became apparent that CC may be responsible for up to 5% of the cases investigated for chronic, watery diarrhea [2]. The disease affects middle-aged and elderly women with a peak incidence around 65 years and a female:male ratio of approximately 7:1 [3]. Over the last few years, several complications of CC have been reported [4], and some of these have been related to the impaired integrity of the colonic wall. Lesions have been described ranging from surface epithelial cell sloughing [5] to sub-mucosal "dissections" [6] and colonic wall tears after endoscopic instrumentation [7]. In a systematic review, Wickbom and co-workers postulated that the impaired compliance of the collagen-containing mucosa was responsible for the tearing of the mucosa by air distension during colonoscopy [8]. Mucosal atrophy, another potential cause of impaired colonic compliance, was neither reported in these cases nor described as a complication of CC [3,5]. Herein we report for the first time the development of an almost fully established mucosal atrophic lesion in the proximal colon of a patient with long standing CC.

## Case presentation

**Figure 1:**
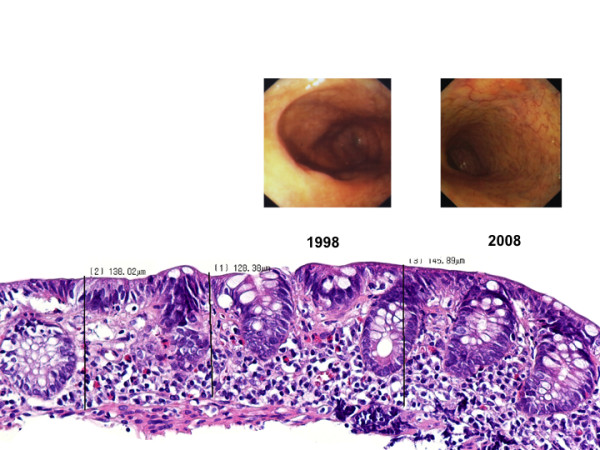
**The 2008 endoscopic image displays a pale, translucent mucosal region from the cecum with prominent a submucosal vascular pattern indicating advanced atrophy**. For comparison the endoscopic image form 1998 displays a normal aspect of cecal mucosa. Please note, the digitally reproduced instant camera image from 1998 displays a somewhat lower resolution. The corresponding histology (hematoxilin and eosin staining) corroborates the endoscopic impression. The height of the cecal mucosa (138 μm [range: 128 - 146 μm]) was dramatically reduced in 2008. Almost no significant sub-epithelial collagen band thickening could be demonstrated in the atrophic cecal mucosa.

**Figure 2:**
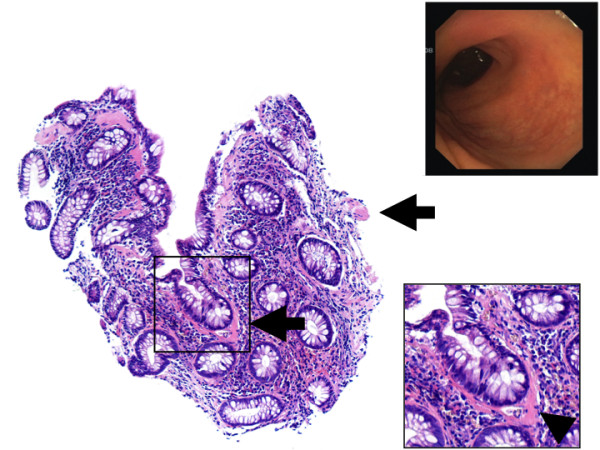
**The normal-appearing rectal mucosa from 2008 is shown**. A small part of the muscular mucosal layer is indicated by the arrowhead at the right side of the low-magnified image. In contrast, extensive collagen band thickening could be seen in the rectal biopsies (arrowhead in the high-magnified image of the sub-epithelium beneath a vertically-cut crypt). Of note, amyloidosis was excluded with special staining (results not shown).

**Figure 3:**
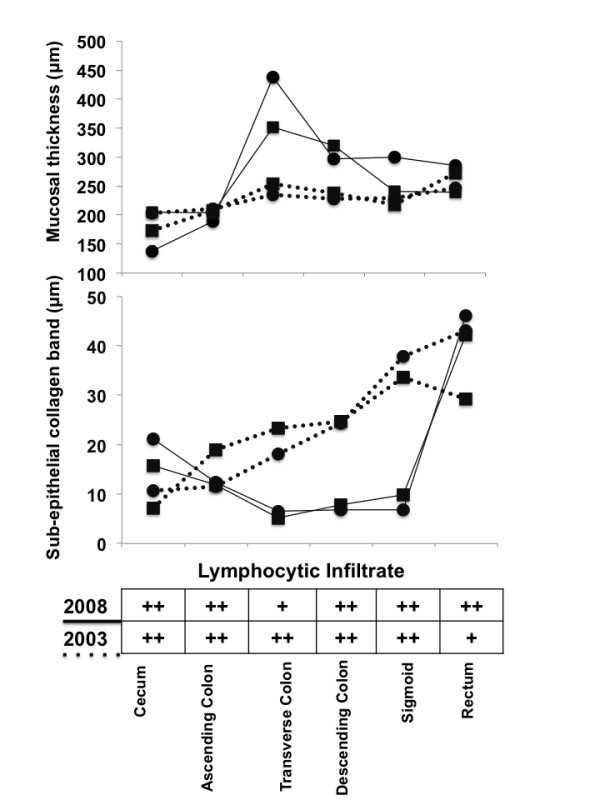
**The mucosal height and the thickness of the sub-epithelial collagen band are shown as assessed by two observers who were blinded to the aim of the study (observer ratings indicated as black squares and dots)**. The results obtained in specimens from the 2003 colonoscopy are connected with dotted lines and those from the 2008 procedure with continuous lines. The semi-quantitative assessment of the lymphocytic infiltrate is shown as table at the bottom of the figure. The results on the semi-quantitative analysis of intraepithelial plasma cells, eosinophils and neutrophils are not shown as they parallel the results for the lymphocytic infiltrate.

A 51-year-old female with a 20-year history of chronic diarrhea was admitted to our gastroenterological rehabilitation center in 2008. Her stool consistency was described as pulpy, and neither blood nor mucus was reported. Her weight (body mass index (BMI): 19 kg/m^2^) had remained constant during the prior months. The physical examination revealed no further specific findings. Routine laboratory tests were within normal values with the exception of decreased serum ferritin levels measured at 9.1 ng/ml (normal: 25 to 120 ng/ml). The diagnosis of CC had been established 10 years prior in 1998 at our center. At the time of diagnosis, the patient's weight was 43 kg (BMI: 16.2 kg/m^2^). She had a history of radioiodine therapy for hyperthyroidism. Three years prior, invasive tubulo-ductal breast cancer (pT1c, grade I-II) was diagnosed in her left breast, and a breast saving R0 resection was performed. Postoperative radiotherapy followed by anti-estrogen therapy with leuporelin and tamoxifen was delivered. Family history was notable for one of her three sisters who had developed Crohn's disease. After the diagnosis of CC was made, she was initially treated with Plantago ovata. In 2002, the patient's weight decreased again by 4 kg to 44 kg (BMI: 16.6 kg/m^2^). One year later, in 2003, a diagnosis CC was corroborated by endoscopic findings at another center and treated with budesonide for two years, resulting in a weight gain of approximately 4 kg. Budesonide was then gradually switched to mesalamine due to the development of osteoporosis. The presence of celiac disease was excluded with normal duodenal biopsies and negative serologic testing. During her inpatient rehabilitation, the third colonoscopy was performed. Parts of the endoscopic and histologic results are shown in Figures 1 and 2. In summary, an advanced mucosal atrophic lesion was diagnosed in the proximal colon, and typical signs of CC were still present. In addition, we examined the reports and endoscopic images obtained from the colonoscopies in 1998, 2003 and 2008. Two pathologists re-analyzed all available biopsies independently and blinded to the aims of the study. The 2003 specimens revealed a medium height of 188 μm of the cecal and a 260 μm height of the rectal mucosa, while 172 μm and 262 μm were measured in the corresponding 2008 specimens (Figure 3). Signs of mucosal atrophy were not present on the histological-reports or protocols from the 1998 colonoscopy nor were they apparent in the available photographic documentation of the 1998 procedure (Figure 1). Budesonide, calcium and vitamin D were prescribed, and the patient was released in a subjectively improved clinical condition with a goal to taper budesonide as fast as possible due to her developing osteoporosis.

## Discussion

We have described a patient with greater than a 10-year history of CC who developed segmental mucosal atrophy involving the ascending colon while sparing the terminal ileum and the remaining segments of the colon. Although, we were not able to obtain a clear-cut and internationally accepted definition of mucosal atrophy, the presence of advanced atrophy in the cecal region of our patient is beyond any reasonable doubt (Figure 1, 2008 endoscopic image and corresponding histology on the bottom). According to established histological textbooks and also according to our own long-time experience, the normal mucosal thickness is between 400-500 μm [9]. In our opinion, these data correspond well to our morphometric finding with a median 188 μm mucosal height in the overtly "atrophic" cecum (Figure 3). However, from Figure 3 it also appears that the apparent mucosal height in the non-atrophic rectum as shown in Figure 2 was rather low with a median height of 262 μm in 2008. This may be explained by that in areas with relatively intact mucosa standard biopsies with limited penetration may not allow for a reliable assessment of mucosal heights beyond a certain threshold.

To our knowledge, such a development of advanced colonic mucosal atrophy has not been documented in the CC literature thus far [3]. Prior to this report, microscopic signs of atrophy in the mucosal lining of the colon were described in only one CC patient by Otegbayo and co-workers [10]. With respect to the time course of the development of mucosal atrophy we observed almost stable mucosal heights comparing cecal and rectal biopsies from 2003 and 2008 (Figure 3). This suggests that cecal atrophy may have evolved before 2003. On the other hand, the 1998 photo documentation (Figure 1) and the endoscopical and histological reports do not support presence of clinically apparent cecal atrophy in 1998. Please note, that comparing the 1998 and 2008 endoscopical images in Figure 1 is somewhat compromised by different resolutions owned to digital reproducing the 1998 original instant camera image. So one may be tempted to pin the start of atrophy to sometime between 1998 and 2003. As the biopsies from 1998 were not available for re-analysis, we cannot rule out that incipient histological signs of cecal atrophy may have been present already at that time. However, it seems to be more likely that evolvement of clinical relevant mucosal atrophy may be better described as late-term sequela of CC.

As expected the number of intraepithelial lymphocytes (IELs) was increased in all parts of the colon (Figure 3). Notably, in ulcerative colitis, Crohn's colitis and infectious colitis IELs are usually not increased. However, a lymphocytic colitis-like pattern in Crohn's disease has been described and may precede the eventual diagnosis of Crohn's disease [11]. In our case the initial symptoms go back into the year 1998, i.e. a 12-year-follow-up. In three endoscopic and histological examinations, the clinico-pathological features of Crohn's disease were not fulfilled, by this rendering the diagnosis of Crohn's disease highly unlikely. In addition, we excluded other concurrent diseases associated with segmental mucosal atrophy, such as ischemic colitis. Although not in the colon, advanced mucosal atrophy has been observed in the duodenum and ileum of patients with CC and celiac disease [12]. However, our patient's duodenal and ileal biopsies did not display any signs of mucosal atrophy. In addition, her serologic tests for celiac disease were negative.

A chance occurrence would be another explanation for the presence of mucosal atrophy with CC in our patient. This type of bias is always relevant for case reports, especially if findings that display a high prevalence are reported in patients with rare diseases. However, after excluding patients with inflammatory bowel disease, the population based prevalences of CC and advanced mucosal atrophy of the proximal colon are comparatively low [3]. Thus, the probability of a random coincidence of these entities seems to be small, although not nil. Regarding the rather profound mucosal atrophy observed in our patient (Figure 1, 2008 endoscopical image and histology), it is noteworthy to mention that additional more extensive damage of the colonic mucosa, such as ulcerations, have also been described in CC patients. Some observations indicate a possible relationship with the concomitant use of medications, such as non-steroidal anti-inflammatory drugs [13]. However, our patient took neither nonsteroidal anti-inflammatory drugs nor did she receive chemotherapy or drugs postulated to be associated with CC or mucosal atrophy [14,15]. Beside drug-induced aggravation of mucosal damage, several other observations indicate a more profound impairment of the colonic wall integrity in some patients with CC. Rarely, sub-mucosal dissections have been described probably alongside the collagen band, [6] and Wickbom and colleagues reviewed a total of 12 patients with mucosal tears and CC [8]. Interestingly, in 10 patients, the mucosal lacerations involved the ascending or the transverse colon, the same exact segments that we observed in the advanced mucosal atrophy in our patient.

Considering that the histopathological abnormalities in CC are believed to be most prevalent in the proximal colon and that the sigmoid colon and rectum may be spared in up to 73% of cases, [16] it has been speculated, that the integrity of the proximal colonic wall may be predominantly compromised owing to the extent of sub-mucosal collagen deposition [5,8]. Although, we were not able to identify any relation between the inflammatory cellular infiltrates and mucosal atrophy in the 2003 and 2008 specimens, we have clearly demonstrated that the sub-epithelial collagen band thickening was consistently described to be maximal in rectal biopsies from all three colonoscopies in our patient (Figure 2). This was corroborated by the morphometric re-analysis of the biopsies from 2003 and 2008 (Figure 3). Additionally, despite there being areas of less collagen band thickening for many years, the cecum and ascending colon were the segments where we observed the maximum atrophy of the colonic mucosa (Figure 1 and 3). This mismatch does not support a direct relationship between the extent of collagen deposition and the development of atrophy in our patient. Rather, our findings foster the assumption, also shared by others [17], that at least sometimes additional luminal factors in combination with the collagen deposits probably influence the activity of CC and thereby propagate mucosal atrophy and colonic wall friability in susceptible patients.

## Conclusion

Our observation introduces the mucosal atrophy of the proximal colon as a novel candidate complication that may be associated with long-standing CC.

## Consent

Written informed consent was obtained from the patient to publish this case report and accompanying images. A copy of the written consent is available for review by the Editor-in-Chief of this journal.

## Abbreviations

CC: collagenous colitis; BMI: body mass index; IELs: intraepithelial lymphocytes

## Competing interests

The authors declare that they have no competing interests.

## Authors' contributions

MM wrote the pathology aspects of the manuscript, provided important input for the interpretation of the histological findings and helped select the histologic specimens that were appropriate for presentation. SW cared for the patient, performed the third colonoscopy, collected the clinical data and prepared the initial presentation of the case. BB prepared the histologic samples and the histologic photographs in Figure 1 and provided important details about their interpretation. H-PF supervised MM with regard to the pathological interpretation and provided fruitful input for the pathological sections of the discussion. CR conducted the entire project, developed the concept and wrote the manuscript. All authors approved the final version of the manuscript.

## Pre-publication history

The pre-publication history for this paper can be accessed here:

http://www.biomedcentral.com/1471-230X/11/114/prepub
